# Donor-dependent immunomodulatory effects of human dental pulp stem cells on macrophages

**DOI:** 10.3389/fimmu.2026.1798470

**Published:** 2026-07-02

**Authors:** Vitor Rodrigues da Costa, Olívia Fonseca Souza, Michelli Ramires Teixeira, Anderson Lucas Alievi, Melissa Martins de Oliveira, Cristina Mary Orikaza, Ana Flávia Popi, Irina Kerkis, Rodrigo Pinheiro Araldi

**Affiliations:** 1 Postgraduate Program in Structural and Functional Biology, Escola Paulista de Medicina of the Universidade Federal de São Paulo (EPM/UNIFESP), São Paulo, Brazil; 2 Instituto Butantan, São Paulo, Brazil; 3 Department of Microbiology, Immunology and Parasitology, Escola Paulista de Medicina of the Universidade Federal de São Paulo (EPM/UNIFESP), São Paulo, Brazil; 4 BioDecision Analytics Ltda., São Paulo, Brazil

**Keywords:** allogenic human dental pulp stem cells (hDPSC), immunomodulation, immunopotency, macrophage, mesenchymal stromal cell (MSCs)

## Abstract

**Background:**

Human dental pulp stem cells (hDPSCs) are a mesenchymal stromal cell (MSC)–like population with emerging therapeutic relevance, yet their immunomodulatory behavior remains incompletely defined.

**Methods:**

Here we examine how hDPSCs from three independent human donors modulate macrophages differentiated from the THP-1 and U-937 monocytic cell lines. Using co-culture systems, we assess cytokine secretion profiles and macrophage surface marker expression by multiplex immunoassays and flow cytometry.

**Results:**

Co-culture revealed donor- and lineage-specific modulation of key cytokines, including TNF-α, IL-10, IL-6, IL-1β, CXCL10, and IL-1RA, while surface marker analysis indicated minimal macrophage polarization. Notably, hDPSCs exhibited transcriptional changes in inflammation-related genes upon interaction with macrophages.

**Conclusions:**

Together, these findings reveal that hDPSC immunomodulatory activity is highly context-dependent and donor-specific, highlighting the importance of donor-aware functional assays for potency assessment and standardization of human cell-based therapies.

## 1 Introduction

Mesenchymal stromal cells, also referred as medicinal signaling cells (MSCs) (1), possess remarkable regenerative and immunomodulatory properties. They are capable of regulating both the innate and adaptive immune responses, making them a promising therapeutic approach for the treatment of autoimmune, inflammatory, and degenerative diseases (2, 3). Consequentely, MSCs have become one of the most well-studied cell products for cell-based therapies (4, 5).

Although the immunomodulatory properties of allogenic MSCs have been widely recognized, their mechanism of action (MoA) remain incompletely understood. In addition, the impact of inherent biological variations, such as those arising from MSCs derived from distinct donors, remains poorly characterized (6, 7), and represents a major regulatory barrier to successfully achieve the clinical translation. Multiple factors, including tissue origin, route of administration, manufacturing processes, and host characteristics can influence MSC potency (7–9). Accordingly, the FDA (10) recommends the use of *in vitro* bioactivity assays to demonstrate the clinical potential of advanced cell therapy products (10, 11). In this context, the immunomodulatory activity of candidate cell therapy products has been investigated using *in vitro* co-culture assays involving MSCs and immune cells (12, 13).

MSCs can be isolated from numerous adult tissues, among them human dental pulp stem cells (hDPSCs) have gained increasing attention recently. Human DPSCs have an ectodermal origin and derive from the neural crest (14, 15). They share key characteristics to conventional MSCs and meet most of the minimal criteria defined by the International Society for Cell & Gene Therapy (ISCT), including: (i) plastic-adhesion capacity when maintained in standard culture conditions, (ii) differentiation capability into osteoblasts and chondroblasts *in vitro*, (iii) the positive expression (>95%) of surface molecules CD73, CD90 and CD105 and, (iv) the lack of expression of markers CD11b, CD19, CD34, CD45 and HLA-DR (<2%) (16). Also, hDPSCs exhibit regenerative and immunomodulatory properties and potential for therapeutic applications, being considered a MSC-like cell (17–19). Notably, hDPSCs uniquely express 394 protein-coding genes that are not expressed in other conventional MSC populations, highlighting their distinct and specialized therapeutic profile (20). While preclinical studies have demonstrated the efficacy of hDPSCs cell therapy in multiple disease models (17), the donor-dependent variability in the immunomodulatory behavior of allogenic hDPSCs remains insufficiently explored, representing a critical barrier to their standardization as therapeutic products.

In this framework, this study investigated differences in the immunomodulatory properties of hDPSCs from different donors *in vitro*. The evaluation included culture of hDPSCs within macrophage models derived from the THP-1 and U-937 cell lines (21, 22). Cytokine secretion and mRNA expression analysis was conducted to assess how donor-specific biological differences and cellular context could influence hDPSC immunopotential.

## 2 Materials and methods

### 2.1 Isolation and culture of hDPSCs

The allogenic human dental pulp stem cells (hDPSC) were isolated from deciduous teeth of three healthy children aged between 6 and 12 years, with all procedures conducted after the signing of the Informed Consent Form, as previously described by Kerkis et al. (23). The manufacturing process of these cells is registrated under US patent number US20160184366A1 and licensed by Cellavita Pesquisas Científicas Ltd., a Brazilian biopharmaceutical company based in São Paulo. The company currently produces these cells at a large scale under Good Manufacturing Practices (GMP) as required by the Brazilian Health Regulatory Agency (Anvisa) for advanced therapy products, as detailed in previous studies (24, 25). These cells comprise the active component of the NestaCell^®^ product (24, 25).

Briefly, the hDPSCs were cultivated up to the fifth passage in Dulbecco’s modified Eagle’s medium (DMEM)/Ham’s F12, supplemented with 15% fetal bovine serum, 100 U/mL penicillin, 100 µg/mL streptomycin, 2 mM L-glutamine and 2 nM nonessential amino acids (all from Gibco, Carlsbad, CA, USA) (23).

Given that each batch of NestaCell^®^ is derived from a different donor and that variation in immunomodulatory responses may occur due to the unique transcriptional profile of each batch or donor, this study investigated, in a comparative manner, the immunomodulatory capability of different batches (donors) of NestaCell^®^, which were identified in this paper as hDPSC-1, −2, and −3.

### 2.2 Characterization of hDPSCs by flow cytometry and cell differentiation

Characterization of hDPSCs as MSC-like occurred using criteria established by the International Society for Cell & Gene Therapy (ISCT) using flow cytometry and cell differentiation assay (16). For flow cytometry, the following fluorochrome-conjugated primary antibodies were used:anti-CD11b BB515 (BD Bioscience, California, USA, reference code 564517), anti-CD19 APC (BD Bioscience, California, USA, reference code 555415), anti-CD34 APC (BD Bioscience, California, USA, reference code 555824), anti-CD45 BB515 (BD Bioscience, California, USA, reference code 564585), Anti-CD73 BB515 (BD Bioscience, California, USA, reference code 565110), Anti-CD90 APC (BD Bioscience, California, USA, reference code 559869), anti-CD105 APC (BD Bioscience, California, USA, reference code 562408), anti-HLA-DR BB515 (BD Bioscience, California, USA, reference code 564516), and the respective isotype controls (BD Bioscience, California, USA, reference code 555751 and 564416).

For surface markers analysis, cell density was adjusted to 0.5x10^6^ cells/mL followed by incubation with primary antibodies for 30 minutes. Next, cells were washed with PBS 1x and analyzed on a flow cytometer BD Accuri™ C6 Plus Flow Cytometer (BD Bioscience, USA). The analysis was performed using FlowJo 10.8.1 software (BD Bioscience, USA).

For chondrogenic differentiation, cells were cultured with Basal Stem Pro medium (Thermo Fisher Scientific, Carlsbad, USA, reference code A10069-01) supplemented with chondrogenic supplement (Thermo Fisher Scientific, Carlsbad, USA, reference code A10064-01). After 14 days of culture, cells were fixed, stained with alcian Blue dye (Sigma-Aldrich, Saint Louis, USA, reference code B8438) and photodocumented to access chondrogenic differentiation micromass formation. Imaging was performed using the Eclipse Ts2-FL photomicroscope (Nikon, Japan, Tokyo) at magnification of 40x.

For osteogenic differentiation, the cell culture medium was replaced with Basal Stem Pro medium (Thermo Fisher Scientific, Carlsbad, USA) supplemented with osteogenic supplement (Thermo Fisher Scientific, Carlsbad, USA, reference code A10066-01) when the cells reached 50% confluence. After 21 days, the cells are fixed, stained with alizarin red dye (Sigma-Aldrich, Saint Louis, USA, reference code: A5533) and photodocumented to detect osteogenic differentiation through foci of mineralization. Imaging was performed using the Eclipse Ts2-FL photomicroscope (Nikon, Japan, Tokyo), at 40x magnification.

### 2.3 Culture of monocytes and macrophages (THP-1 and U-937 cell lineages)

Two commercial monocytic cell lineages, THP-1 (monocytes derived from peripheral blood of a patient with acute monocytic leukemia, BCRJ 0234) and U-937 (monocytes isolated from pleural effusion of lymphoma, BCRJ 0242), were employed to assess the immunomodulatory potential of hDPSCs. Both cell lines were sourced from the Rio de Janeiro Cell Bank (BCRJ - The Rio de Janeiro Cell Bank) and were authenticated by short-tandem repeat (STR)-PCR analysis to confirm the absence of cross-contamination (data not shown).THP-1 and U-937 cell lines were cultivated in Roswell Park Memorial Institute (RPMI) 1640 medium, supplemented with 10% fetal bovine serum (FBS) and 1% penicillin/streptomycin solution (all from Gibco, Carlsbad, USA) with HEPES. Cells were cultivated at 37°C, with a humidified atmosphere of 5% CO_2_.

### 2.4 Differentiation of THP-1 and U-937 monocytes into unstimulated macrophages (M0)

A total of de 1.041 X 10^5^ monocytes from THP-1 and U-937 were seeded per cm^2^ in a 6 or 12-well plate containing 5 mL or 2 mL of complete medium containing 100 ng/mL (THP-1) and 20 ng/mL (U-937) of phorbol-12-myristate-12-acetate (PMA) (Sigma-Aldrich, Saint Louis, USA), for differentiation into unstimulated (resting) macrophages (also known as M0 macrophages), as previously reported (21). Cell adherence was used as an indicator of successful differentiation into macrophages. After 48 hours, nonadherent cells were aspirated and the medium of both cell lineages was replaced by complete medium without PMA.

### 2.5 Analysis of the immunomodulatory potential of the hDPSCs on THP-1 and U-937 M0 macrophages

The immunomodulatory potential of hDPSCs was evaluated using bioactivity assays on macrophage populations focusing on cytokine production and immune phenotyping of macrophages in co-culture systems (13).

For this, hDPSCs were detached using the CTS™TrypLE™ Select (Gibco, Reference code: A12859-01, Denmark), and a total of 0.208 x 10^5^ hDPSCs/cm^2^ were seeded per well in 6- or 12-well plates along with 1.041 X 10^5^ of previous differentiated M0 macrophages (derived from THP-1 or U-937 cell lines) per cm^2^, corresponding to a 1:5 ratio of hDPSCs to macrophages. In dose-dependence, experiments varying cell ratios were tested, including 1:5, 1:10, 1:50 and 1:100 hDPSCs/macrophages. The viability of hDPSCs was observed using the trypan blue exclusion assay. After 48 hours, the culture medium was collected and centrifuged for 5 minutes at 500 g at room temperature to remove non-adherent cells from the supernatant. The resulting supernatant was then immediately cryopreserved at -80 °C to preserve the integrity of secreted cytokines and chemokines (for subsequent dosage using LEGENDplex method). Following this, cells were subjected to further analysis.

### 2.6 Cytokine quantification

Cytokines in the culture media of treated and untreated unstimulated macrophages derived from THP-1 and U-937 cell lineages co-cultured with the hDPSC were quantified using the Human Macrophage/Microglia Panel (13-plex) kit (Biolegend, reference code 740503). This kit enables the simultaneous measurement of cytokines including IL-12p70, TNF-α, IL-6, IL-1β, IL-12p40, IL-23, IFN-γ, CXCL10, IL-4, IL-10, Arginase, CCL17, and IL-1RA, following the fabricant instructions. For cytokine analysis, 25 μL of supernatants from the mono- or co-culture were mixed with 25 μL of particle suspension and 25 μL of Assay Buffer in a V-bottom 96-well plate. The final mixture was incubated for two hours at room temperature with constant agitation to prevent particle sedimentation. Following incubation, the samples were centrifuged at 250xg (RCF), and the supernatant was carefully removed to avoid disturbing the bead pellet. The beads were then washed with 1x Wash Buffer, and then 25 μL of detection antibodies were added to each well. The plates were incubated for one hour at room temperature with constant agitation, followed by the addition of 25 μL of SAPE (Streptavidin-Phycoerythrin) to each well. The final suspension was incubated for 30 minutes at room temperature with agitation.

After the final incubation, the samples were centrifuged again at 250xg (RCF), and the supernatant was carefully discarded. The beads were then washed with 1x Wash Buffer resuspended in the same buffer. Cytokine levels were quantified by acquiring the beads on the BD FACSCanto II Cell Analyzer (BD Bioscience, USA), which had been calibrated prior to use. Data was analyzed using the LEGENDplex™ Data Analysis Software (Biolegend, USA). The cytokine assay was performed with three biological replicates in two independent experiments per donor and per condition.

### 2.7 Gene expression analysis

To assess which cell type modulated the cytokine secretion profile in the co-culture, cells were sorted using the Aria III FACS (BD Biosciences). THP-1 or U-937 macrophages were identified as CD45^+^CD90^-^ events, while hDPSCs were identified as CD45^-^CD90^+^ events. A minimum purity threshold of 97% was required for subsequent analyses (**Supplementary Figure 1**). Total RNA was then extracted using TRIzol™ Reagent (Invitrogen, Carlsbad, CA, USA, reference code 15596026). 1μg of total RNA was used for cDNA synthesis with the High-Capacity cDNA Reverse Transcription kit (Applied Biosystems, Carlsbad, EUA, reference code 4368814), following the manufacture instructions. Quantitative PCR (qPCR) reaction was performed using PowerUp™ SYBR™ Green Master Mix (Applied biosystems, Foster City, CA, USA, reference code A25742) on QuantStudio™ 3 Real-Time PCR System (Applied biosystems, Foster City, CA, USA). *HPRT1* was used as the reference gene. Cycling conditions and primers are outlined in **Supplementary Tables 1** and **2**, respectively. The cytokine qPCR assay was performed with three biological replicates in two independent experiments using hDPSCs derived from one-donor (hDPSCs-1).

To perform an exploratory evaluation of inflammation-related gene expression in hDPSCs cultured in monoculture or after co-culture with THP-1 or U-937 macrophages, a PCR array assay was performed using the TaqMan™ Array 96-Well Plate Human Inflammation (Applied Biosystems, reference code 4414205). This plate enables the simultaneous evaluation of 92 genes related to the inflammatory process. Assays were conducted according to the manufacturer’s instructions, using: 2 ng of cDNA per reaction, 10 µL of TaqMan Gene Expression Master Mix (2X) (Thermo Fisher Scientific, Carlsbad, USA), and diethyl pyrocarbonate (DEPC) water (Thermo Fisher Scientific, Carlsbad, USA) to complete the reaction to a final volume of 20 µL. The cycling conditions used in this assay are described in **Supplementary Table 1**.

For the Human Inflammation qPCR Array analysis, reference genes *18S*, *HPRT1*, *GAPDH*, and *GUSB* were used. Genes with a delta Ct (dCt) value less than 13 and classified as amplified by the analysis software (status = Amp) were considered expressed. For the construction of graphs, genes that did not meet these criteria were assigned a Ct value of 38.

The fold-change (FC) of inflammation related genes from hDPSCs derived from co-cultures was calculated in relation to hDPSCs control group (monoculture). Based on the FC values obtained, genes with an FC of less than 0.75 were considered negatively regulated (or downregulated – DRG), and genes with an FC greater than 1.25 were considered positively regulated (or upregulated – URG). Genes with FC values between 0.75 and 1.25 were considered equally expressed (or equally expressed genes – EEG). The qPCR-Array screening assay was performed with one pooled biological sample in one experiment, using hDPSCs derived from one-donor (identified as hDPSCs-1).

### 2.8 Functional enrichment analysis

To identify the genes responsible to modulate the higher number of pathways, functional enrichment analyses were conducted to investigate biological processes and molecular pathways associated with the identified genes using an over-representation analysis (ORA) approach. This method assesses the enrichment of specific gene sets based on the presence or absence of genes of interest, without considering their expression levels. Enrichment results were obtained for Gene Ontology (GO) terms, covering biological processes and molecular functions, and for biological pathways using the Enrichr web platform. Enrichr integrates multiple well-established databases, including Reactome 2024, WikiPathways 2024, BioPlanet 2019, Kyoto Encyclopedia of Genes and Genomes (KEGG) 2021, Elsevier Pathway Collection, Panther 2016, NCI-Nature 2016, HumanCyc 2016, BioCarta 2016, and MSigDB Hallmark 2020. To ensure statistical robustness, only pathways with a false discovery rate (FDR) < 0.05 were considered significantly enriched, following the recommendations of Mathur et al. (26).

### 2.9 Immunophenotyping by flow cytometry and gating strategy

To analyze macrophage polarization, considering that macrophages can polarize into different phenotypes depending on the environmental stimulus, flow cytometry was performed using the pro-inflammatory macrophage markers CD64, CD80, CD282 and CD284, or anti-inflammatory macrophage markers CD163 and CD206. Cells were first adjusted to a density of 1.0 × 10^5^ cells/mL and then were sorted for staining with the LIVE/DEAD™ Fixable Aqua Dead Cell Stain (Thermo Fisher Scientific, Carlsbad, EUA, reference code L34957), according to the fabricant instructions. To block Fc receptors, Fc Receptor Binding Inhibitor Polyclonal Antibody (Thermo Fisher Scientific, Carlsbad, EUA, reference code 14-9161-73) was added for 15 minutes at 4°C.

Primary antibodies used included anti-CD45 (BD Bioscience, California, USA, reference code 564585), anti-CD64 (Invitrogen, Carslabd, USA, reference code 25-0649-42), anti-CD80 PE (BD Bioscience, California, USA, reference code 557227), anti-CD163 AF647 (Invitrogen, Carslabad, USA, reference code 63-1639-42), anti-CD206 BV421 (BD Bioscience, California, USA, reference code 564062), anti-CD282 APC (Invitrogen, Carslabd, USA, reference code 17-9922-42) and anti-CD284 PE (BD Bioscience, California, USA, reference code 564215). The incubation was performed for 30 minutes at 4°C in the dark. After, cells were washed with PBS 1x and fixed with 4% formaldehyde for 15 minutes.

All the samples were acquired in a previously calibrated BD FACSCanto II Cell Analyzer (BD Bioscience, USA) cytometer and the results were analyzed using the FlowJo 10.8.1 software (BD Bioscience, USA).

First, cell doublets and cellular debris were excluded. Live macrophages (CD45^+^ Live/Dead^-^ cells) were separated from hDPSCs (CD45^-^ cells), and the macrophages were then analyzed for the surface markers CD64, CD80, CD163, CD206, CD282 and CD284. The gating strategy for this analysis is shown in **Supplementary Figure 2**.

### 2.10 Statistical analysis

Statistical analyses of mRNA relative expression and Immunophenotyping were performed using the *t*-Student test (for comparisons between two groups), while one-way ANOVA followed by Tukey’s *post hoc* test (for comparisons among two or more groups) were used to cytokine analysis both with a significance threshold set at 5%. The assumption of normality (Shapiro-Wilk test), and homogeneity of variances test (Levene’s test), were checked prior to conducting the tests, p-values < 0.05 were considered non-normal and non-homogeneous. For samples with unequal variances, the Welch correction was applied together with Games-Howell *post-hoc*. Pearson’s Correlation was used to dose-dependence assay. All the analyses were carried out using Jamovi v2.2.5 software and graphs were generated using the Graph Prism 8 software (GraphPad Software, Inc, California, USA). Results are presented as Mean ± Standard Deviation (SD) with p-values indicating statistical significance: p < 0.001; ***, p < 0.01; **, p < 0.05, * and ns, not significant.

### 2.11 Ethics statement

All procedures conducted in this study were approved by the Research Ethics Committee of the Federal University of São Paulo (CEP-UNIFESP; CEP n° 0498/2023; CAAE: 70149923.2.0000.5505).

## 3 Results

### 3.1 Characterization of hDPSCs

**Figure 1:**
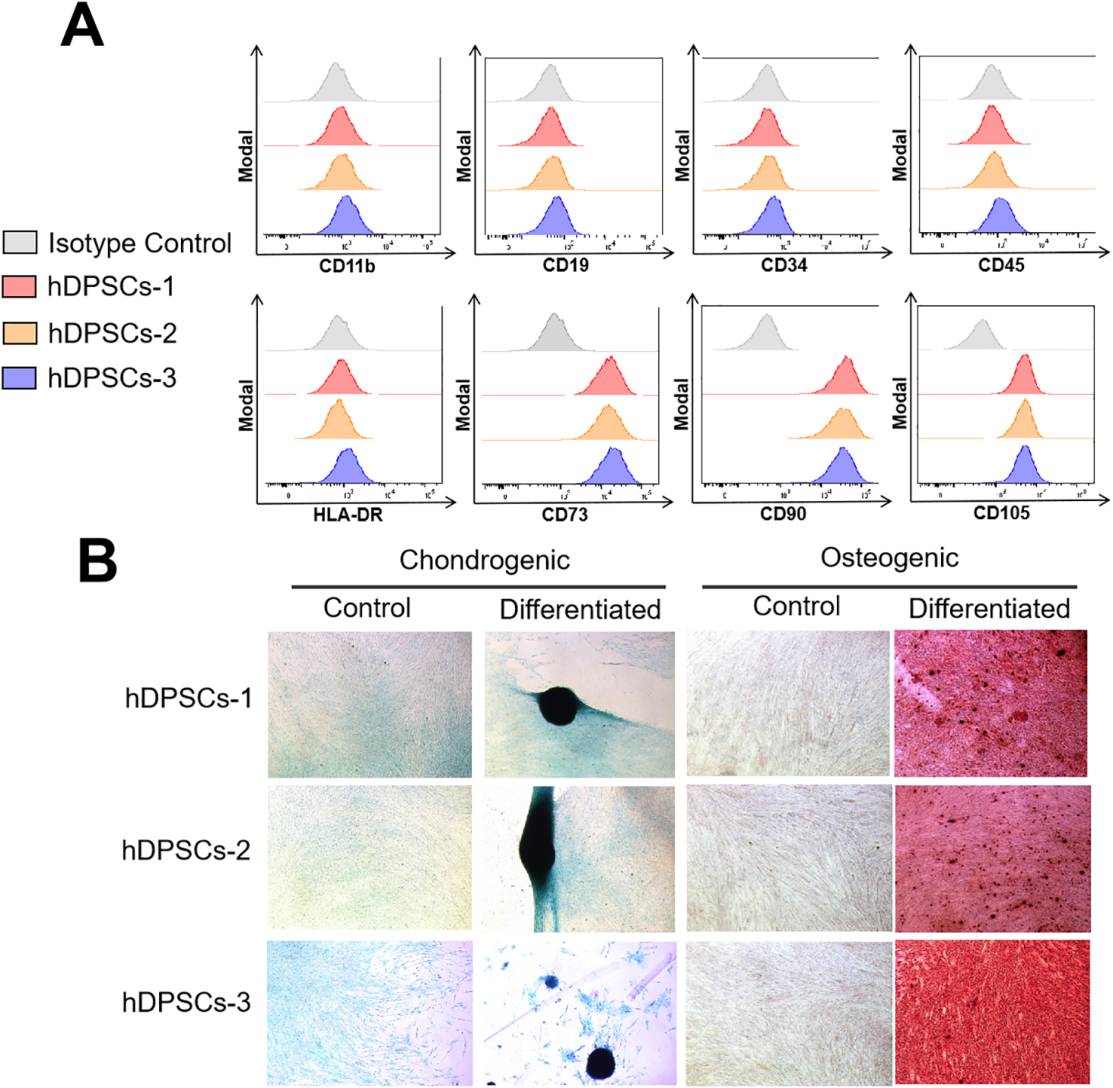
Characterization of Human Dental Pulp Stem Cells (hDPSC) derived from different donors. **(A)** Flow cytometry analysis of positive markers of MSCs, such as CD73, CD90 and CD105; and negative markers, such as CD11b, CD19, CD34, CD45 and HLA-DR. Gray histograms represent isotype controls, while red, orange and blue histograms represent hDPSCs-1, -2 and -3, respectively. **(B)** hDPSCs have plastic adherence and fusiform morphology and exhibited mesodermal multidifferentiation capacity for chondrogenesis (alcian blue staining) and osteogenesis (alizarin red staining). Control hDPSCs were not submitted to the induction of differentiation.

To confirm that the hDPSCs retained the MSC-like phenotype, we evaluated the expression of canonical MSC markers and their differentiation potential according to ISCT criteria was performed (16). All three hDPSCs batches exhibited the expected immunophenotype, characterized by negligible expression of hematopoietic markers CD11b, CD19, CD34, CD45, and HLA-DR (< 2% of cells) and strong expression of CD73, CD90 and CD105 (> 95% of cells) (**Figure 1A**), consistent with previous reports (24).

Morphologically, hDPSCs display a spindle-shaped and plastic-adherent phenotype. After 21 days of lineage-specific induction, cells successfully differentiated into chondrocytes, as evidenced by alcian blue staining of glycosaminoglycans (that are abundant in cartilage extracellular matrix), and into osteocytes, demonstrated by alizarin red staining of mineralized matrix, indicating osteogenic differentiation (**Figure 1B**).

### 3.2 Allogenic hDPSCs derived from different donors differently modulate M0 macrophages derived from THP-1 or U-937 cells

Given that the immunomodulatory capacity of hDPSCs may vary according to donor, as reported for other conventional MSCs populations (7), here it was investigated how hDPSCs from different donors modulate macrophages derived from the THP-1 and U-937 cell lines, focusing on cytokine secretion profiles.

**Figure 2:**
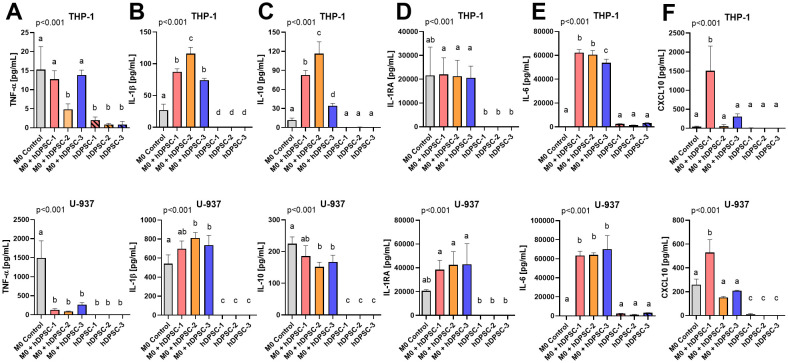
Cytokine secretion profiles of THP-1 and U-937 macrophages after co-culture with hDPSCs from different donors. THP-1 (top row) and U-937 (bottom row) M0 macrophages were cultured alone or co-cultured with human dental pulp stem cells (hDPSCs) derived from three different donors (hDPSC-1, -2, and -3). Supernatants were collected after 48 hours, and cytokine levels were quantified. Box plots show the secretion of **(A)** TNF-α, **(B)** IL-1β, **(C)** IL-10, **(D)** IL-1RA, **(E)** IL-6, and **(F)** CXCL10. The data illustrate donor-dependent and cell line-specific modulation of both pro- and anti-inflammatory cytokines following hDPSC–macrophage interaction. Differences among experimental groups were analyzed by one-way ANOVA followed by Tukey’s *post hoc* test; distinct lowercase letters indicate statistically significant differences (*p* < 0.05). The cytokine assay was performed with three biological replicates in two independent experiments per donor and per condition.

Overall, hDPSCs from different donors exerted distinct immunomodulatory effects, and both donor variability and macrophage cell line identity influenced cytokine responses. Among the three hDPSC batches tested, only hDPSC-2 significantly reduced TNF-α secretion in THP-1 macrophages co-cultures, while hDPSC-1 and hDPSC-3 had no significant effect. In contrast, all three hDPSC donors markedly suppressed TNF-α production by U-937 macrophages (**Figure 2A**).

For IL-1β, co-culture with any hDPSC batch led to a significant increase in IL-1β secretion by THP-1 macrophages. In contrast, co-culture of U-937 macrophages with hDPSCs-1 did not significantly alter IL-1β levels compared with their respective monocultures (**Figure 2B**). All hDPSCs batches enhanced the secretion of IL-10 in THP-1 co-cultures; however, hDPSCs-3 showed a markedly reduced capacity to increase IL-10 compared with hDPSCs-1 and hDPSCs-2 batches. Conversely, hDPSs-2 and hDPSCs-3 reduced IL-10 levels in U-937 co-cultures, while hDPSCs-1 did not significantly affect IL-10 production, when compared to U937 macrophage monoculture (**Figure 2C**).

IL-1RA secretion by THP-1 macrophages remained unaffected following co-culture with hDPSCs. All hDPSC donors showed a trend toward increased IL-1RA levels in U-937 macrophages (**Figure 2D**). Co-culture with any hDPSC batch increased IL-6 secretion in both THP-1 and U-937 macrophages supernatant co-cultures (**Figure 2E**). CXCL10 levels were selectively modulated by hDPSC-1, which increased its secretion in both cell lines; hDPSC-2 and hDPSC-3 had no significant effect (**Figure 2F**). The variability measures of multiplex cytokine data are listed in **Supplementary Table 3**. In general, the cytokine regulation showed a strong correlation with the number of hDPSCs in co-culture (**Supplementary Figure 3**).

**Figure 3:**
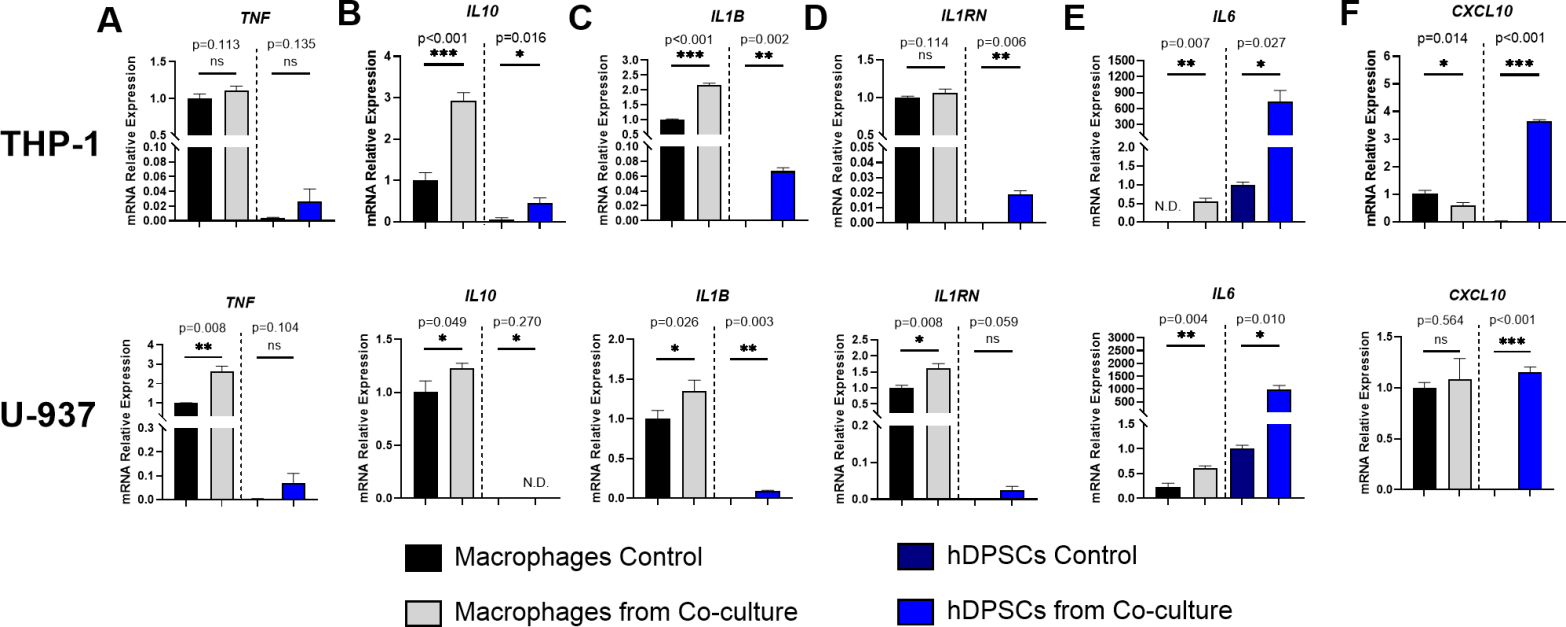
Cytokine gene expression in macrophages and hDPSCs after co-culture. Relative mRNA expression of **(A)** *TNF*, **(B)** *IL10*, **(C)** *IL1B*, **(D)** *IL1RN*, **(E)** *IL6* and **(F)** *CXCL10* determined by qPCR. The upper panels show gene expression in THP-1-derived macrophages and the lower panels in U-937-derived macrophages, after co-culture with hDPSCs-1. For each cytokine, expression was evaluated separately in macrophages (left side of the dashed line; black = monoculture control, grey = macrophages after co-culture with hDPSCs) and in hDPSCs (right side of the dashed line; dark blue = hDPSCs monoculture, blue = hDPSCs after co-culture). Gene expression was normalized to *HPRT1* housekeeping gene. The *t*-Student test was used to evaluate the mRNA relative expression by qPCR with 5% of significance level, with p-values indicated above brackets (*p < 0.05, **p < 0.01, ***p < 0.001; ns, not significant). Data representative of two independent experiments using hDPSCs derived from one-donor.

Following the analysis of the macrophage-associated cytokine secretion, we sought to identify the primary cellular source of the detected cytokines. For this, we selectively evaluated the cytokine mRNA expression on macrophages and hDPSCs-1 cells isolated from co-cultures and compared them with their respective monoculture controls. The qPCR analysis suggested that CXCL10 and IL-6 were predominantly produced by hDPSCs-1, as the expression of *IL6* and *CXCL10* dramatically increased following the co-culture with both THP-1 and U-937 macrophages. In contrast, TNF-α, IL-10, IL-1β and IL-1RA appeared to be primarily macrophage-derived, as their mRNA levels were substantially upregulated in both THP-1 and U-937 macrophages after co-culture. Consistently, hDPSCs-1 exhibited minimal expression of *TNF*, *IL10*, *IL1B*, and *IL1RN* compared with macrophages (**Figure 3**). The variability measures of cytokine qPCR data are listed in **Supplementary Table 4**.

Both M0 macrophage models and all three hDPSC donors produced high levels of the anti-inflammatory cytokine Arginase, exceeding the detection limit of the assay used. In contrast, IL-12p40, IL-12p70, IL-4, IL-23, IFN-γ, and CCL17 were barely detectable, with most cytokine levels falling below the sensitivity threshold of the method in both mono- and co-cultures. Consequently, these cytokines were considered undetectable in THP-1 and U-937 M0 macrophages. A summary of these findings is provided in **Supplementary Figure 4**.

In summary, these findings highlight the functional heterogeneity among hDPSC donors, demonstrating that the immunomodulatory impact on macrophages is both donor- and cell line-dependent. Such variability reinforces the need for rigorous donor screening and functional validation when considering hDPSCs for immunoregulatory or therapeutic applications.

Analysis of macrophage surface markers revealed limited phenotypic shifts. No significant changes were observed in CD64, CD80, CD206, or CD282 in either THP-1 or U-937 macrophages after co-culture with the hDPSCs-1. However, THP-1 macrophages exhibited increased expression of the anti-inflammatory marker CD163, whereas U-937 macrophages showed reduced levels of the pro-inflammatory marker CD284; the reciprocal markers remained unchanged in the respective cell lines (**Supplementary Figure 5**). These findings suggest that hDPSC-mediated immunomodulation predominantly affects cytokine secretion rather than global macrophage phenotypes, with selective cell type-specific effects.

### 3.3 Reciprocal regulation: macrophages induce gene expression changes in hDPSCs

Given the bidirectional nature of the hDPSC–macrophage interaction, in which macrophages increased IL-6 and CXCL10 secretion by hDPSCs (**Figure 3**), we performed an exploratory screening of inflammation-related genes, using a 92-gene qPCR array. This analysis was conducted using pooled RNA obtained from the hDPSCs-1 cells following co-culture with THP-1 or U-937 M0 macrophages. Therefore, it should be interpreted as hypothesis-generating.

**Figure 4:**
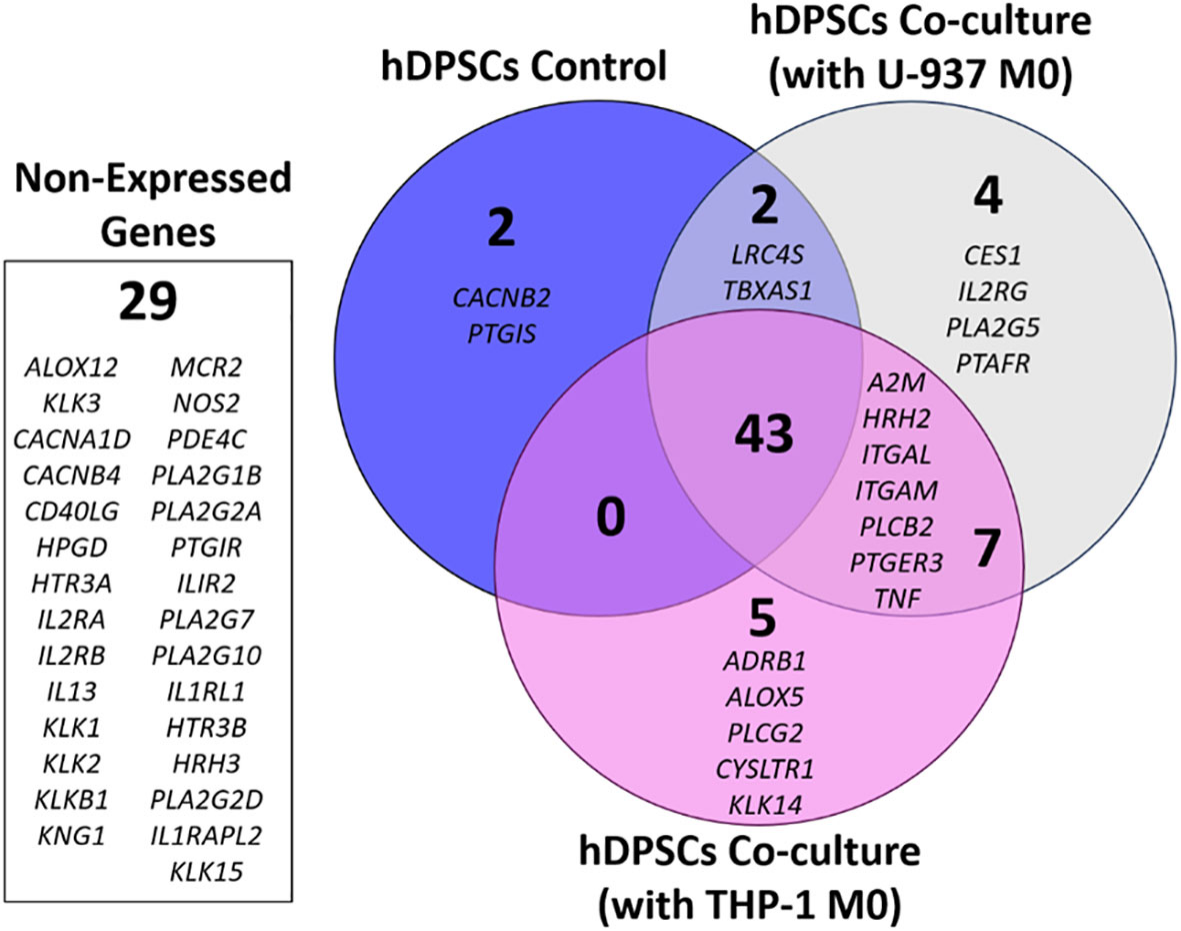
Analysis of Inflammation-related genes in hDPSCs after co-culture with THP-1 or U-937 M0 macrophages. Venn diagram represents the number of inflammation-associated genes commonly or exclusively expressed in the hDPSCs-1 monoculture and in hDPSCs-1 derived from co-cultures with THP-1 or U-937 macrophages. Data representative of one pooled biological sample in one experiment, using hDPSCs derived from one-donor.

The results showed that, from the 92 genes present in the array, 29 were not expressed in hDPSCs control neither the co-cultured groups. While 2 genes (*CACNB2* and *PTGIS*) were exclusively expressed in control hDPSCs, 4 genes (*CES1*, *IL2RG*, *PLA2G5* and *PTAFR*) were expressed just in hDPSCs derived from co-culture with U-937 macrophages, and 5 genes (*ADRB1*, *ALOX5*, *PLCG2*, *CYSLTR1* and *KLK14*) were only expressed in hDPSCs derived from co-culture with THP-1 macrophages. In addition, 2 genes (*LRC4S* and *TBXAS1*) were expressed in hDPSCs control and derived from U-937 co-cultures, but not in hDPSCs derived from THP-1 M0 co-cultures. In contrast, 7 genes (*A2M*, *HRH2*, *ITGAL*, *ITGAM*, *PLCB2*, *PTGER3* and *TNF*) gained expression when hDPSCs were co-cultured with THP-1 and U-937 M0 macrophages. 43 genes were collectively expressed in the hDPSCs control and co-cultured with THP-1 and U-937 macrophages. These results are summarized in the Venn Diagram represented in **Figure 4**. Altogether, these observations indicate that THP-1 and U-937 macrophages selectively modulate hDPSCs gene expression, suggesting the reciprocal and context-dependent nature of their interaction. However, given the exploratory nature of this single-donor dataset, these findings should be considered preliminary.

### 3.4 Macrophage-specific modulation of inflammation-related gene expression in hDPSCs

Analysis of the 43 genes co-expressed across all hDPSCs groups (monocultures and co-cultures with THP-1 or U-937 M0 macrophages), revealed a strong concordance in the expression patterns, with similar upregulation and downregulation of inflammation-related genes in hDPSCs co-cultured with either THP-1- or U-937-derived macrophages. However, while most genes exhibited consistent regulation across both macrophage conditions, some genes showed differential modulation in one co-culture group, and others were oppositely regulated between the two macrophage lines. Notably, the magnitude of fold changes in gene expression varied significantly depending on the macrophage cell line used, highlighting the macrophage-specific nature of the regulatory response (**Table 1**) However, confirmation in independent donors is required.

**Table 1:** Relative expression of inflammation-related genes mutually expressed in hDPSCs co-cultured with THP-1 or U-937 M0 macrophages.

Genes	hDPSCs co-cultured with			
	THP-1 M0		U-937 M0	
	Fold-change	Status	Fold-change	Status
*PTGFR*	0,712	DRG	0,629	DRG
*CACNA1C*	0,696	DRG	0,573	DRG
*VCAM1*	0,482	DRG	0,313	DRG
*PLCB4*	0,451	DRG	0,028	DRG
*PDE4B*	0,388	DRG	0,718	DRG
*ADRB2*	0,266	DRG	0,617	DRG
*BDKRB1*	0,170	DRG	0,470	DRG
*BDKRB2*	0,167	DRG	0,412	DRG
*PTGER2*	0,125	DRG	0,164	DRG
*CASP1*	0,337	DRG	1,136	EEG
*ANXA5*	0,383	DRG	0,766	EEG
*NR3C1*	0,517	DRG	1,148	EEG
*ANXA3*	0,931	EEG	0,750	EEG
*PLCE1*	0,967	EEG	0,170	DRG
*MAPK3*	1,105	EEG	1,203	EEG
*LTA4H*	1,138	EEG	0,982	EEG
*PTGS1*	4,669	URG	0,852	EEG
*HRH1*	2,059	URG	1,082	EEG
*TNFRSF1A*	1,958	URG	1,130	EEG
*MAPK8*	1,899	URG	1,073	EEG
*ANXA1*	1,433	URG	0,783	EEG
*TNFSF13B*	1,021	EEG	2,244	URG
*LTB4R*	0,816	EEG	1,440	URG
*ICAM1*	24,846	URG	92,043	URG
*CD40*	9,150	URG	1,276	URG
*PTGIR*	7,657	URG	21,784	URG
*PTGS2*	7,318	URG	32,363	URG
*ITGB2*	4,519	URG	3,165	URG
*PDE4D*	4,330	URG	3,609	URG
*PLCG1*	3,087	URG	2,413	URG
*NFKB1*	2,131	URG	2,025	URG
*MAPK1*	2,052	URG	1,601	URG
*LTB4R2*	2,034	URG	2,279	URG
*TNFRSF1B*	1,909	URG	4,002	URG
*PLA2G4C*	1,661	URG	6,422	URG
*MAPK14*	1,569	URG	1,439	URG
*IL1R1*	1,495	URG	2,187	URG
*ITGB1*	1,351	URG	2,323	URG
*PDE4A*	1,306	URG	1,359	URG
*TBXA2R*	1,291	URG	1,365	URG
*CACNA2D1*	4,196	URG	0,474	DRG
*PLCD1*	0,740	DRG	2,405	URG
*PLCB3*	0,504	DRG	2,020	URG

To generate hypothesis regarding potential regulatory pathway, we focused on genes with the highest pathway connectivity that were consistently up- or down-regulated in hDPSCs derived from both co-culture conditions. The results showed that *MAPK1, TNF, MAPK14* and *MAPK8* are the commonly URG, while the *CACNA1C*, *PLCB4*, *VCAM1* and *ADRB2* are the commonly DRG on hDPSCs after co-culture with THP-1 or U-937 macrophages (**Supplementary Figure 6**). Based on known gene functions, the results of upregulated and downregulated genes indicates that after co-culture with macrophages, hDPSCs can show a feedback mechanism to dampen excessive inflammatory signaling consistent with anti-inflammatory adaptation. The activation of MAPK family can indicates the activation of hDPSCs, while the decrease in *CACNA1C*, *PLCB4* expression (which encodes the Cav1.2 L-type calcium channel and the phospholipase C beta 4, respectively) can impact negatively on calcium intracellular signaling. Besides that, the decrease in *VCAM1* expression might suggest an impairment on therapeutic capacity (27, 28). However, because these data derive from a pooled sample from a single donor and lack functional validation, these interpretations remain speculative.

Overall, this targeted transcriptional screening provides preliminary insights into potential pathways involved in macrophage–hDPSC crosstalk and serves as a basis for future mechanistic studies.

## 4 Discussion

Allogenic hDPSCs, a distinct therapeutic cell population, are gaining recognition for their high immunomodulatory potential in regenerative medicine and therapeutic potential in pre-clinical models (17–19). In special, the allogenic hDPSCs at the fifth passage (P5) used in this study have been explored in Phase I/II clinical trials for Huntington’s disease (https://clinicaltrials.gov/ Identifiers NCT02728115, NCT03252535 and NCT04219241). In this context, this study employed unpolarized THP-1 and U-937 macrophages to explore whether hDPSCs can influence macrophage phenotype in absence of predefined polarization, given the complex inflammatory environment in Huntington’s disease characterized by monocyte and macrophage plasticity and heterogeneity, and not a fixed pro-inflammatory state throughout (29), this approach was used to examine the direct potential of allogenic hDPSC to modulate macrophage phenotype *de novo*.

It was observed that the hDPSCs used in this work, derived from three different batches (donors), share characteristics in common with other typical MSCs, including most of the minimal defined criteria by the ISCT for MSC. These characteristics did not predict the immunomodulatory effects of hDPSCs over macrophages in this system. This observation is consistent with previous reports indicating that the proposed MSCs markers do not reliably predict the therapeutic potency (30, 31).

Specific studies examining the immunomodulatory properties of hDPSCs from different donors remain limited. Our findings demonstrate that allogeneic hDPSCs derived from distinct donors, even when manufactured under strict Good Manufacturing Practice (GMP) conditions and following a standardized production protocol and same characterization, can induce markedly divergent cytokine secretion profiles in macrophages sharing the same genetic background. This underscores that intrinsic donor-dependent factors represent a major and unavoidable source of biological heterogeneity that cannot be eliminated through standardized cell processing alone. Consistent with observations in bone marrow– and umbilical cord–derived MSCs, donor-related variables such as age, sex, epigenetic landscape, and genetic background can substantially influence the immunomodulatory potential of these cells (8, 32, 33). These intrinsic differences may have important implications for clinical translation: while GMP manufacturing ensures safety and consistency in basic quality attributes, it does not guarantee uniform biological activity across donors. Approaches such as donor pre-screening, pooling cells from multiple donors, or donor selection based on functional markers have been proposed as potential strategies to address this variability, but their effectiveness requires further investigation. Therefore, rather than demonstrating direct clinical implications, these results support the growing notion that functional assays may be required to complement standard MSC characterization.

Given the inter-batch variability observed, it is noteworthy that a hDPSC derived from the same donor could distinctly modulate M0 macrophages derived from THP-1 or U-937 cell lines, both in terms of cytokine secretion and immunophenotype. This modulation was evidenced by the downregulation of the pro-inflammatory marker CD284 (TLR4) on U-937 macrophages and the upregulation of the anti-inflammatory marker CD163 on THP-1 macrophages. These findings suggest that macrophages lineage and cellular context may influence hDPSCs-macrophage crosstalk *in vitro*. It is well known that MSCs modify its immunomodulatory activity after the MSCs licensing by macrophage secretome and environment cytokines (13, 34–36). Consistent with this concept, the co-culture was associated with increased IL-6 and CXCL10 secretion by hDPSCs and by the markedly differential expression of inflammation-related genes in hDPSCs after co-culture with THP-1 or U937 M0 macrophages. This included proposed key genes to the immunomodulatory potential of typical MSCs, such as *PTGS2*, which are responsible for PGE2 synthesis, one of the most important immunomodulatory factors secreted by MSCs (37–39), suggesting that hDPSCs adjust their immunological profile according to their environment. However, functional studies would be required to confirm these mechanisms.

The analysis of the differential expressed genes (DEG) showed that *MAPK1, TNF, MAPK14* and *MAPK8* are the URG on hDPSCs after co-culture with THP-1 or U-937 macrophages that modulate the higher quantity of pathways. Although studies about the activity of *MAPK1* and *MAPK14* (which encode the proteins ERK2 and p38, respectively) in hDPSCs are still limited. There are studies showing that the expression of *MAPK1* and *MAPK14* are elevated in dental pulp cells from inflamed pulp (e.g. carious/inflamed teeth and LPS induced inflammation). This suggests that *MAPK1* and *MAPK14* are part of the “inflammatory signature” of hDPSCs *in situ* when exposed to an inflammatory environment (40, 41). Also, *MAPK8*, which encodes JNK1 protein, was one of the URG on hDPSCs after co-culture with THP-1 macrophages, which often works in concert with p38 (*MAPK14*) and ERK (*MAPK1/3*) to integrate stress and inflammatory signals (42). Thus, indicating that hDPSCs may be activated after co-culture with the THP-1 or U-937 macrophages. However, in the absence of functional validation, these findings should be interpreted as indicative of potential pathway involvement rather than evidence of pathway activation. Although mRNA levels of *TNF* increased on hDPSCs after co-culture with THP-1 or U-937 macrophages, no increase in TNF cytokine levels were registered in this study, suggesting that the secretion of this cytokine by hDPSCs are limited. However, low levels of TNF can be secreted. In this regard, different studies showed that the TNF signaling through the TNF-R2, in contrast to signaling by TNF-R1, can promote MSC survival, immunomodulatory and pro-regenerative differentiation (43, 44). Indeed, qPCR results show that hDPSCs increased the expression of the *TNFRSF1B*, which encodes TNF-R2 protein, after the co-culture with THP-1 or U-937 macrophages, however, the functional relevance of this finding remains to be determined.

Furthermore, the *CACNA1C*, *PLCB4*, *VCAM1* and *ADRB2* are the DRG on hDPSCs after co-culture with THP-1 or U-937 macrophages that modulates the higher quantity of pathways. As seen in the URG, studies about the role of these genes on hDPSCs are still limited. However, functional analysis of these genes indicated that hDPSCs may exhibit a feedback mechanism to dampen excessive inflammatory signaling, consistent with an anti-inflammatory adaptation. Specifically, activation of the MAPK family suggests hDPSC activation, while the decreased expression of CACNA1C and PLCB4 can negatively impact intracellular calcium signaling, which is critical for the inflammatory response. Besides that, the decrease in *VCAM1* expression might suggest an impairment on therapeutic capacity, since VCAM1^+^ MSCs, derived from chorionic villi and umbilical cord, have been suggested as more immunosuppressive cells (27, 28). Nevertheless, these interpretations remain speculative and require targeted experimental validation.

It is important to note that these interpretations on the gene expression data are speculative and based on the known functions of each gene. It is important to emphasize that these interpretations are inferences which, although grounded in current knowledge of gene function, require experimental validation in future studies.

Altogether, these findings highlight the complex and donor-dependent immunomodulatory effects of hDPSCs on macrophages, which vary across macrophage lineages. These results are hypothesis-generating rather than conclusive. Specifically, they raise the possibility that inflammatory licensing signals from macrophages may influence hDPSC phenotype. Identifying molecules that could polarize hDPSCs toward a desired immunomodulatory profile may, after further validation, inform future translational research.

The present work has limitations: (i) all the experiments were conducted using the macrophage models derived from THP-1 and U937 cell lines, which do not completely reproduce the macrophage biology, phenotypic heterogeneity, functional plasticity, and donor-dependent variability characteristic of primary human macrophages *in vivo*. In this scenario, the immunomodulatory effects of hDPSCs described here should be interpreted with caution when extrapolating to physiological settings. Importantly, the lack of validation in primary monocyte-derived macrophages represents a key limitation of this work. Therefore, future studies using human monocyte-derived macrophages derived from healthy subjects will be necessary to reinforce the immunomodulatory potential of hDPSCs on macrophages and confirm the translational relevance of these findings; (ii) The cell-to-cell contact or paracrine effects of hDPSCs under macrophages were not specifically evaluated in this study. In this sense, previous studies have shown that the hDPSCs immunomodulatory capacity under macrophages is complex, depending concomitantly on cell-to-cell interaction and paracrine mediators, including secreted factors (45); (iii) the study only includes three hDPSCs donors/batches, which may not fully capture the variability seen in a broader donor population. Therefore, future studies should include a larger cohort to improve generalizability. Accordingly, these hypothesis-generating findings support the need for future studies using primary macrophages or monocyte-derived macrophages, larger donor cohorts, a mechanistic dissection of paracrine versus contact-dependent signaling, and the evaluation therapeutic potential of hDPSCs derived from different donors in validated disease models.

## Data Availability

The original contributions presented in this study are publicly available. The datasets generated and analyzed during the current study have been deposited in the Zenodo repository and can be accessed at: https://doi.org/10.5281/zenodo.20739330.

## References

[1] Caplan AI . Mesenchymal stem cells: Time to change the name! Stem Cells Transl Med. (2017) 6:1445–51. doi: 10.1002/sctm.17-0051 28452204 PMC5689741

[2] Shi Y Wang Y Li Q Liu K Hou J Shao C . Immunoregulatory mechanisms of mesenchymal stem and stromal cells in inflammatory diseases. Nat Rev Nephrol. (2018) 14:493–507. doi: 10.1038/s41581-018-0023-5 29895977

[3] Song N Scholtemeijer M Shah K . Mesenchymal stem cell immunomodulation: Mechanisms and therapeutic potential. Trends Pharmacol Sci. (2020) 41:653–64. doi: 10.1016/j.tips.2020.06.009 32709406 PMC7751844

[4] Ramezankhani R Torabi S Minaei N Madani H Rezaeiani S Hassani SN . Two decades of global progress in authorized advanced therapy medicinal products: An emerging revolution in therapeutic strategies. Front Cell Dev Biol. (2020) 8. doi: 10.3389/fcell.2020.547653 33392179 PMC7773756

[5] Iglesias-López C Agustí A Obach M Vallano A . Regulatory framework for advanced therapy medicinal products in Europe and United States. Front Pharmacol. (2019) 10. doi: 10.3389/fphar.2019.00921 31543814 PMC6728416

[6] Gao F Chiu SM Motan DAL Zhang Z Chen L Ji HL . Mesenchymal stem cells and immunomodulation: Current status and future prospects. Cell Death Dis. (2016) 7:e2062. doi: 10.1038/cddis.2015.327 26794657 PMC4816164

[7] Mckinnirey F Herbert B Vesey G McCracken S . Immune modulation via adipose derived mesenchymal stem cells is driven by donor sex *in vitro*. Sci Rep. (2021) 11:12454. doi: 10.1038/s41598-021-91870-4 34127731 PMC8203671

[8] Galipeau J Krampera M Leblanc K Nolta JA Phinney DG Shi Y . Mesenchymal stromal cell variables influencing clinical potency: The impact of viability, fitness, route of administration and host predisposition. Cytotherapy. (2021) 23:368–81. doi: 10.1016/j.jcyt.2020.11.007 33714704 PMC11708105

[9] Kozlowska U Krawczenko A Futoma K Jurek T Rorat M Patrzalek D . Similarities and differences between mesenchymal stem/progenitor cells derived from various human tissues. World J Stem Cells. (2019) 11:347–74. doi: 10.4252/wjsc.v11.i6.347 31293717 PMC6600850

[10] FDA . Potency tests for cellular and gene therapy products. (2011), 1–17. Guidancy for Industry.

[11] Galipeau J Krampera M Barrett J Dazzi F Deans RJ DeBruijn J . International Society for Cellular Therapy perspective on immune functional assays for mesenchymal stromal cells as potency release criterion for advanced phase clinical trials. Cytotherapy. (2015) 18:151–9. doi: 10.1016/j.jcyt.2015.11.008 26724220 PMC4745114

[12] de Wolf C van de Bovenkamp M Hoefnagel M . Regulatory perspective on *in vitro* potency assays for human mesenchymal stromal cells used in immunotherapy. Cytotherapy. (2017) 19:784–97. doi: 10.1016/j.jcyt.2017.03.076 28457740

[13] Saldaña L Bensiamar F Vallés G Mancebo FJ García-Rey E Vilaboa N . Immunoregulatory potential of mesenchymal stem cells following activation by macrophage-derived soluble factors. Stem Cell Res Ther. (2019) 10:58. doi: 10.1186/s13287-019-1156-6 30760316 PMC6375172

[14] De Souza PV Alves FBT Costa Ayub CLSA De Miranda Soares MA Gomes JR . Human immature dental pulp stem cells (hIDPSCs), their application to cell therapy and bioengineering: An analysis by systematic revision of the last decade of literature. Anat Rec. (2013) 296:1923–8. doi: 10.1002/ar.22808 24130093

[15] Nuti N Corallo C Chan BMF Ferrari M Gerami-Naini B . Multipotent differentiation of human dental pulp stem cells: a literature review. Stem Cell Rev Rep. (2016) 12:511–23. doi: 10.1007/s12015-016-9661-9 27240827

[16] Dominici M Le Blanc K Mueller I Slaper-Cortenbach I Marini FC Krause DS . Minimal criteria for defining multipotent mesenchymal stromal cells. The International Society for Cellular Therapy position statement. Cytotherapy. (2006) 8:315–7. doi: 10.1080/14653240600855905 16923606

[17] Yamada Y Nakamura-Yamada S Kusano K Baba S . Clinical potential and current progress of dental pulp stem cells for various systemic diseases in regenerative medicine: A concise review. Int J Mol Sci. (2019) 20:1132. doi: 10.3390/ijms20051132 30845639 PMC6429131

[18] Andrukhov O Behm C Blufstein A Rausch-Fan X . Immunomodulatory properties of dental tissue-derived mesenchymal stem cells: Implication in disease and tissue regeneration. World J Stem Cells. (2019) 11:604–17. doi: 10.4252/wjsc.v11.i9.604 31616538 PMC6789188

[19] Anderson S Prateeksha P Das H . Dental pulp-derived stem cells reduce inflammation, accelerate wound healing and mediate M2 polarization of myeloid cells. Biomedicines. (2022) 10:1999. doi: 10.3390/biomedicines10081999 36009546 PMC9624276

[20] Araldi RP Viana M Avelar Colozza-Gama G Dias Pinto JR Ankol L Valverde CW . Unique transcriptional signatures observed in stem cells from the dental pulp of deciduous teeth produced on a large scale. (2023) 14:72–95. doi: 10.17311/pharmacologia.2023.72.95

[21] Chanput W Peters V Wichers H . THP-1 and U937 cells. In: The Impact of Food Bioactives on Health: *In Vitro* and Ex Vivo Models. Cham, Switzerland: Springer (2015). doi: 10.1007/978-3-319-16104-4_14 29787039

[22] Nascimento CR Rodrigues Fernandes NA Gonzalez Maldonado LA Rossa Junior C . Comparison of monocytic cell lines U937 and THP-1 as macrophage models for *in vitro* studies. Biochem Biophys Rep. (2022) 32:101383. doi: 10.1016/j.bbrep.2022.101383 36420419 PMC9677084

[23] Kerkis I Kerkis A Dozortsev D Stukart-Parsons GC Gomes Massironi SM Pereira LV . Isolation and characterization of a population of immature dental pulp stem cells expressing OCT-4 and other embryonic stem cell markers. Cells Tissues Organs. (2007) 184:105–16. doi: 10.1159/000099617 17409736

[24] Fernandes JMS Pagani E Wenceslau CV Ynoue LH Ferrara L Kerkis I . Phase II trial of intravenous human dental pulp stem cell therapy for Huntington’s disease: a randomized, double-blind, placebo-controlled study. Stem Cell Res Ther. (2025) 16:114. doi: 10.1186/s13287-025-04557-2 40770775 PMC12330078

[25] Wenceslau CV de Souza DM Mambelli-Lisboa NC Ynoue LH Araldi RP Silva J . Restoration of BDNF, DARPP32, and D2R expression following intravenous infusion of human immature dental pulp stem cells in Huntington’s disease 3-NP rat model. Cells. (2022) 11:1664. doi: 10.3390/cells11101664 35626701 PMC9139280

[26] Mathur R Rotroff D Ma J Shojaie A Motsinger-Reif A . Gene set analysis methods: A systematic comparison. Biodata Min. (2018) 11:8. doi: 10.1186/s13040-018-0166-8 29881462 PMC5984476

[27] Liu H Cui D Huangfu S Wang X Yu X Yang H . VCAM-1+ mesenchymal stem/stromal cells reveal preferable efficacy upon an experimental autoimmune encephalomyelitis mouse model of multiple sclerosis over the VCAM-1- counterpart. Neurochem Res. (2025) 50:839–54. doi: 10.1007/s11064-024-04267-w 39613932 PMC11607028

[28] Yang ZX Han ZB Ji YR Wang YW Liang L Chi Y . CD106 identifies a subpopulation of mesenchymal stem cells with unique immunomodulatory properties. PloS One. (2013) 8:e59354. doi: 10.1371/journal.pone.0059354 23555021 PMC3595282

[29] Di Pardo A Alberti S Maglione V Amico E Cortes EP Elifani F . Changes of peripheral TGF-β1 depend on monocytes-derived macrophages in Huntington disease. Mol Brain. (2013) 6:55. doi: 10.1186/1756-6606-6-55 24330808 PMC4029620

[30] Deskins DL Bastakoty D Saraswati S Shinar A Holt GE Young PP . Human mesenchymal stromal cells: Identifying assays to predict potency for therapeutic selection. Stem Cells Transl Med. (2013) 2:151–58. doi: 10.5966/sctm.2012-0099 23362238 PMC3659751

[31] Lv FJ Tuan RS Cheung KMC Leung VYL . Concise review: The surface markers and identity of human mesenchymal stem cells. Stem Cells. (2014) 32:1408–19. doi: 10.1002/stem.1681 24578244

[32] Siegel G Kluba T Hermanutz-Klein U Bieback K Northoff H Schäfer R . Phenotype, donor age and gender affect function of human bone marrow-derived mesenchymal stromal cells. BMC Med. (2013) 11:146. doi: 10.1186/1741-7015-11-146 23758701 PMC3694028

[33] Zhang C Zhou L Wang Z Gao W Chen W Zhang H . Eradication of specific donor-dependent variations of mesenchymal stem cells in immunomodulation to enhance therapeutic values. Cell Death Dis. (2021) 12:357. doi: 10.1038/s41419-021-03644-5 33824286 PMC8024246

[34] Ißleib C Kurz S Scholl S Amberg B Spohn J . Plasticity of proinflammatory macrophages depends on their polarization stage during human MSC immunomodulation—An *in vitro* study using THP-1 and human primary macrophages. Immuno. (2021) 1:396–414. doi: 10.3390/immuno1040036 30654563

[35] Noronha Nc NDC Mizukami A Caliári-Oliveira C Cominal JG Rocha JLM Covas DT . Priming approaches to improve the efficacy of mesenchymal stromal cell-based therapies. Stem Cell Res Ther. (2019) 10:131. doi: 10.1186/s13287-019-1224-y 31046833 PMC6498654

[36] Herger N Heggli I Mengis T Devan J Arpesella L Brunner F . Impacts of priming on distinct immunosuppressive mechanisms of mesenchymal stromal cells under translationally relevant conditions. Stem Cell Res Ther. (2024) 15:123. doi: 10.1186/s13287-024-03677-5 38443999 PMC10916130

[37] Lu D Xu Y Liu Q Zhang Q . Mesenchymal stem cell-macrophage crosstalk and maintenance of inflammatory microenvironment homeostasis. Front Cell Dev Biol. (2021) 9. doi: 10.3389/fcell.2021.681171 34249933 PMC8267370

[38] Vasandan AB Jahnavi S Shashank C Prasad P Kumar A Jyothi Prasanna S . Human mesenchymal stem cells program macrophage plasticity by altering their metabolic status via a PGE 2 -dependent mechanism. Sci Rep. (2016) 6:38308. doi: 10.1038/srep38308 27910911 PMC5133610

[39] Ko JH Kim HJ Jeong HJ Lee HJ Oh JY . Mesenchymal stem and stromal cells harness macrophage-derived amphiregulin to maintain tissue homeostasis. Cell Rep. (2020) 30:3806–20.e6. doi: 10.1016/j.celrep.2020.02.062 32187551

[40] Arora S Cooper PR Friedlander LT Seo B Rizwan SB Rich AM . Potentiality and inflammatory marker expression are maintained in dental pulp cell cultures from carious teeth. Int J Mol Sci. (2022) 23:9425. doi: 10.3390/ijms23169425 36012689 PMC9409171

[41] Kim JH Irfan M Hossain MA Shin S George A Chung S . LPS-induced inflammation potentiates dental pulp stem cell odontogenic differentiation through C5aR and p38. Connect Tissue Res. (2023) 64:741–54. doi: 10.1080/03008207.2023.2218944 37247252 PMC10524681

[42] Vervaeke A Lamkanfi M . MAP kinase signaling at the crossroads of inflammasome activation. Immunol Rev. (2025) 329:e13436. doi: 10.1111/imr.13436 39754394

[43] Beldi G Bahiraii S Lezin C Nouri Barkestani M Abdelgawad ME Uzan G . TNFR2 is a crucial hub controlling mesenchymal stem cell biological and functional properties. Front Cell Dev Biol. (2020) 8. doi: 10.3389/fcell.2020.596831 33344453 PMC7746825

[44] Kelly ML Wang M Crisostomo PR Abarbanell AM Herrmann JL Weil BR . TNF receptor 2, not TNF receptor 1, enhances mesenchymal stem cell-mediated cardiac protection following acute ischemia. Shock. (2010) 33:602–7. doi: 10.1097/SHK.0b013e3181cc0913 19953003 PMC3076044

[45] Maccaferri M Pisciotta A Carnevale G Salvarani C Pignatti E . Human dental pulp stem cells modulate pro-inflammatory macrophages both through cell-to-cell contact and paracrine signaling. Front Immunol. (2024) 15. doi: 10.3389/fimmu.2024.1440974 39450172 PMC11499095

