# Supplementary material for: Donor-dependent immunomodulatory effects of human dental pulp stem cells on macrophages
Source: Front Immunol. 2026 Jul 2;17:1798470. doi: 10.3389/fimmu.2026.1798470 (PMC13372703; doi:10.3389/fimmu.2026.1798470)
Supplement: Supplementary file 1 [file DataSheet1.docx]

**SUPPLEMENTARY MATERIALS**

SUPPLEMENTARY FIGURES


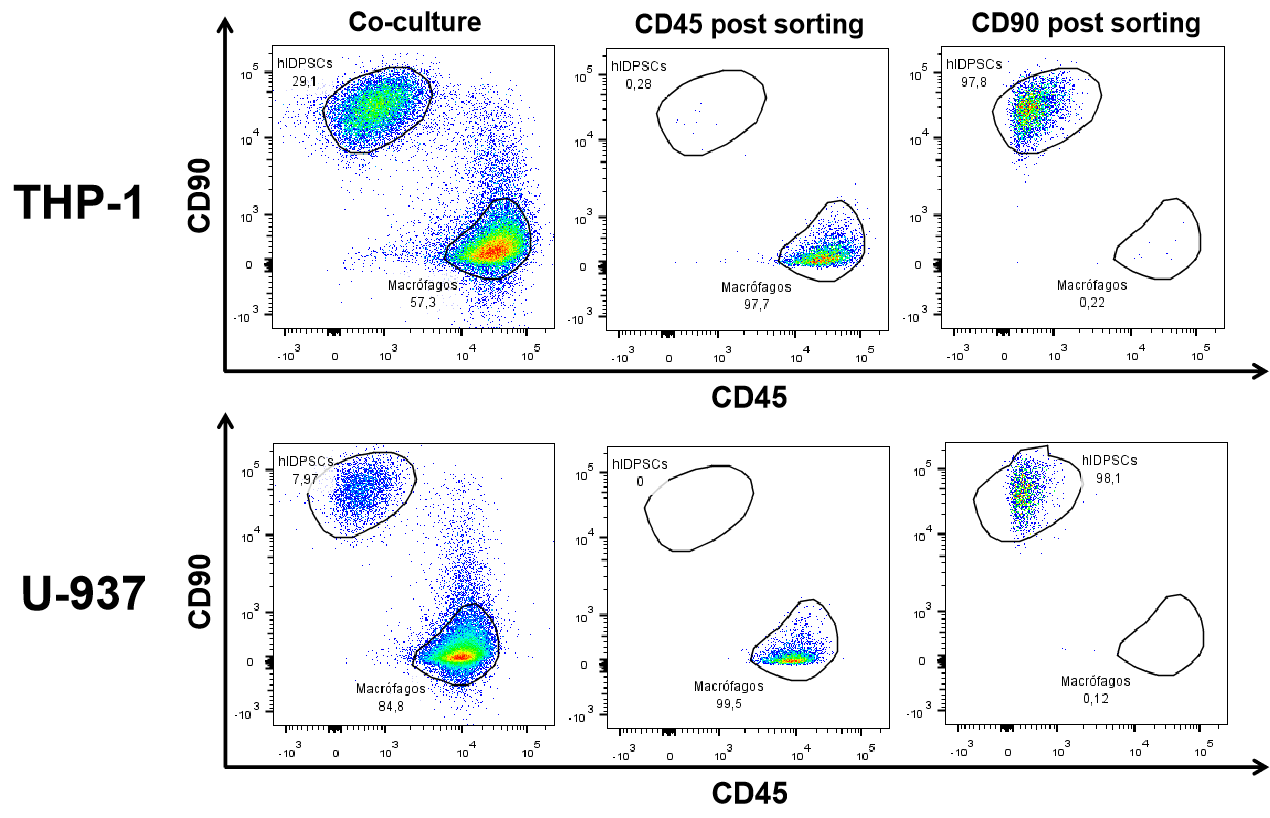


**Supplementary Figure 1. Analysis of the cell separation efficiency of THP-1 and U-937 derived macrophages from hDPSCs after direct co-culture.** Using flow cytometry and the markers CD45 and CD90 to identify macrophages (CD45^+^CD90^-^) and hDPSC (CD45^-^CD90^+^), the efficiency of cell separation performed by the FACS Aria III Cell Sorter was evaluated. The sorting of macrophages and hDPSCs from co-cultures was performed with a purity greater than 97%.


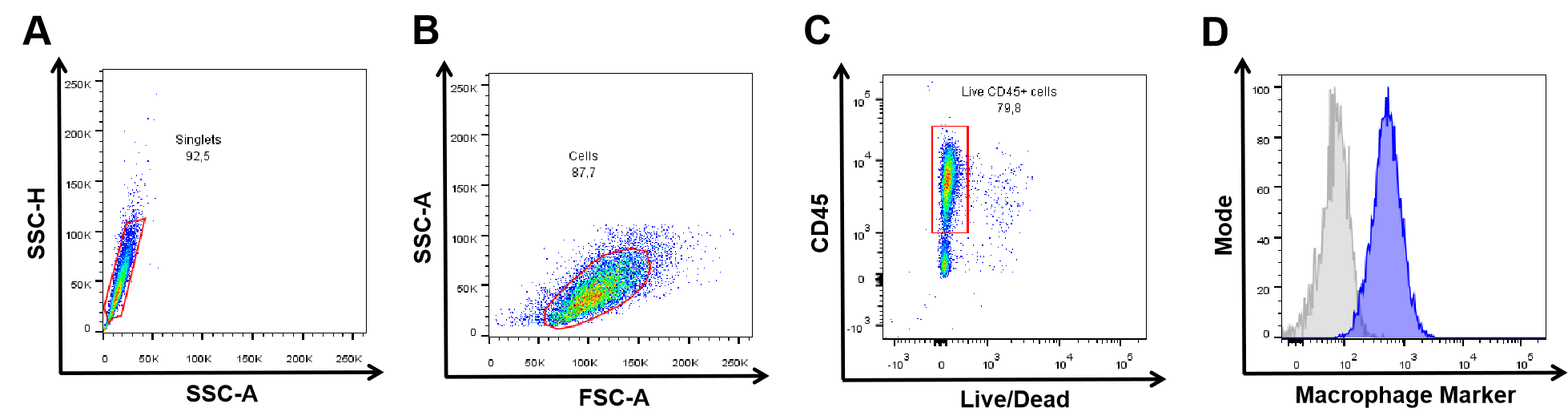


**Supplementary Figure 2. Gating Strategy to evaluate macrophage phenotype**. Initially, (**A**) cell singlets were selected, then (**B**) cellular debris were excluded. After, (**C**) live macrophages (CD45^+^ Live/Dead^-^ events) were separated from hDPSCs (CD45^-^ events), then (**D**) macrophages were analyzed to CD64, CD80, CD163, CD206, CD282 and CD284 surface markers.


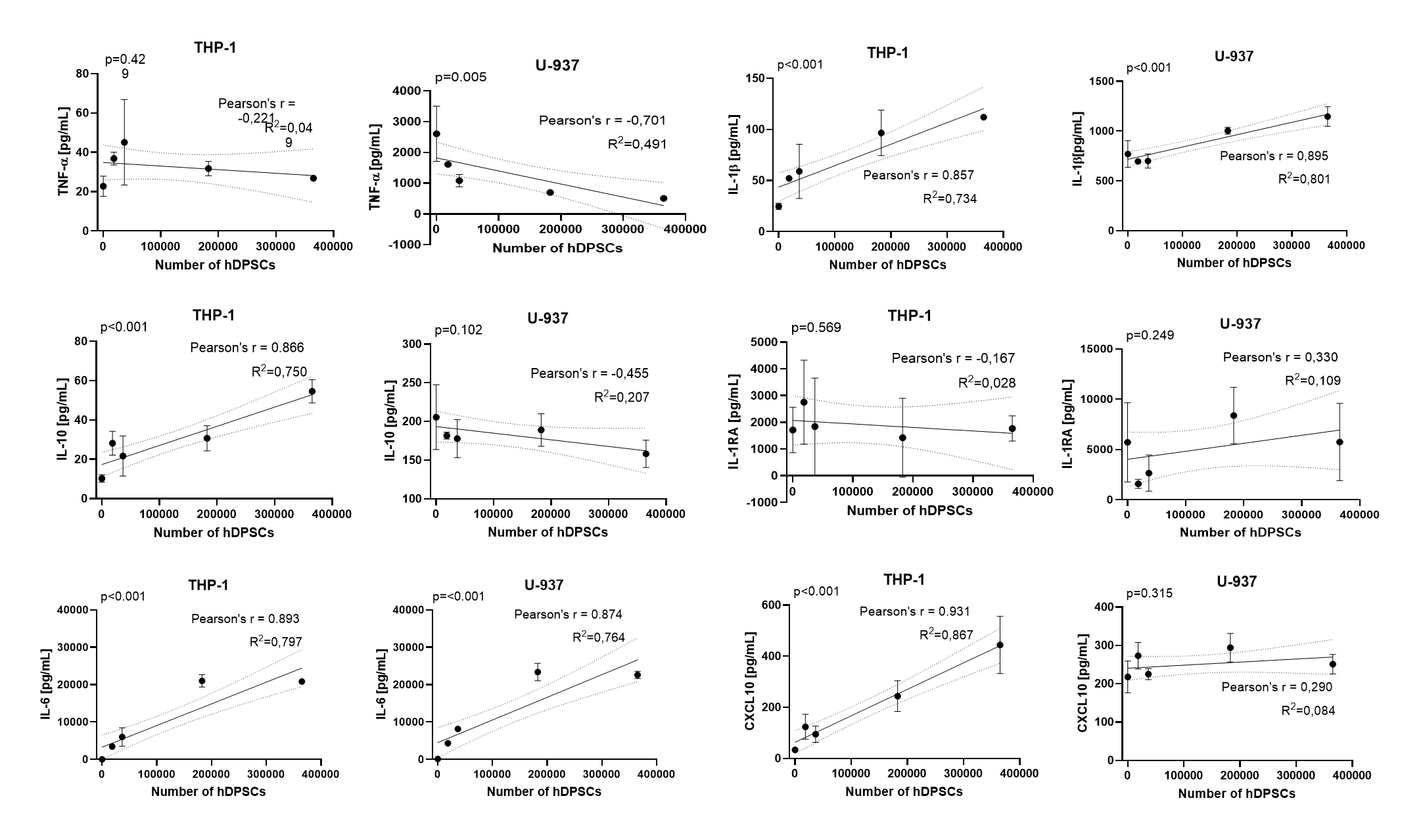


**Supplementary Figure 3. Allogenic hDPSCs modulate THP-1 and U-937 macrophages in a dose dependent manner.** The co-culture of THP-1 or U-937 macrophages with different hDPSCs quantities showed that the secretion of the majority of cytokines are strongly correlated with the quantity of hDPSCs on co-cultures. Pearson’s Correlation was used to dose-dependence assay.


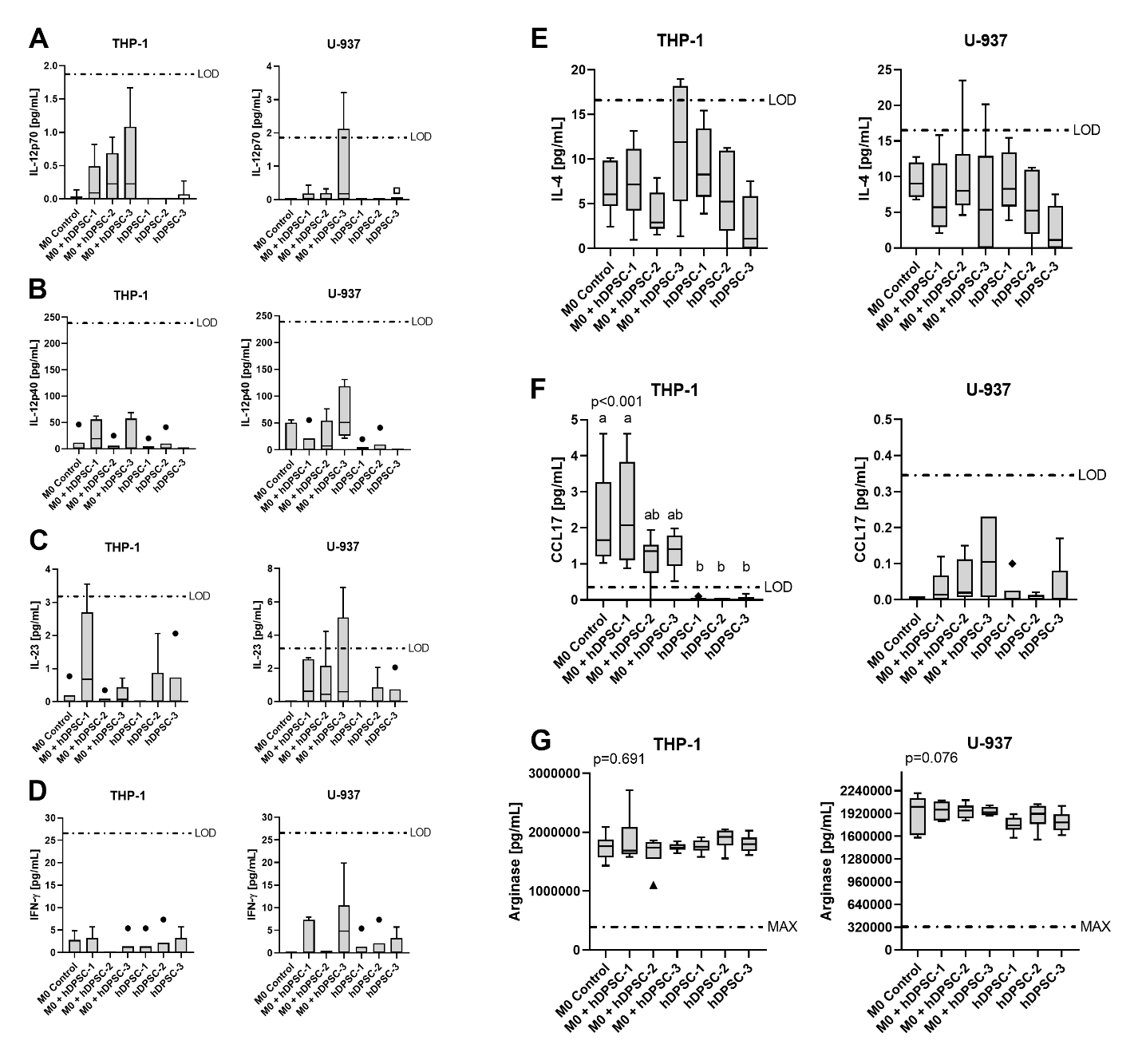


Supplementary Figure 4. Cytokines out of the minimum and maximum limit of quantification. The pro-inflammatory cytokines (A) IL-12p70, (B) IL-12p40, (C) IL-23, (D) IFN-γ, and the anti-inflammatory cytokines (E) IL-4 and (F) CCL17, were below the limit of detection in mono- and co-cultures of hDPSCs with THP-1 or U-937 unstimulated macrophages. In contrast, the anti-inflammatory mediator (G) Arginase was highly secreted in mono- and co-cultures of hDPSCs with THP-1 or U-937 unstimulated macrophages, being out of the maximum limit of detection of the assay. One way ANOVA were analyzed with 5% of significance level. Outliers are represented as figures outside the boxplot. Legend: LOD = Limit of Detection; MAX= maximum limit of detection.


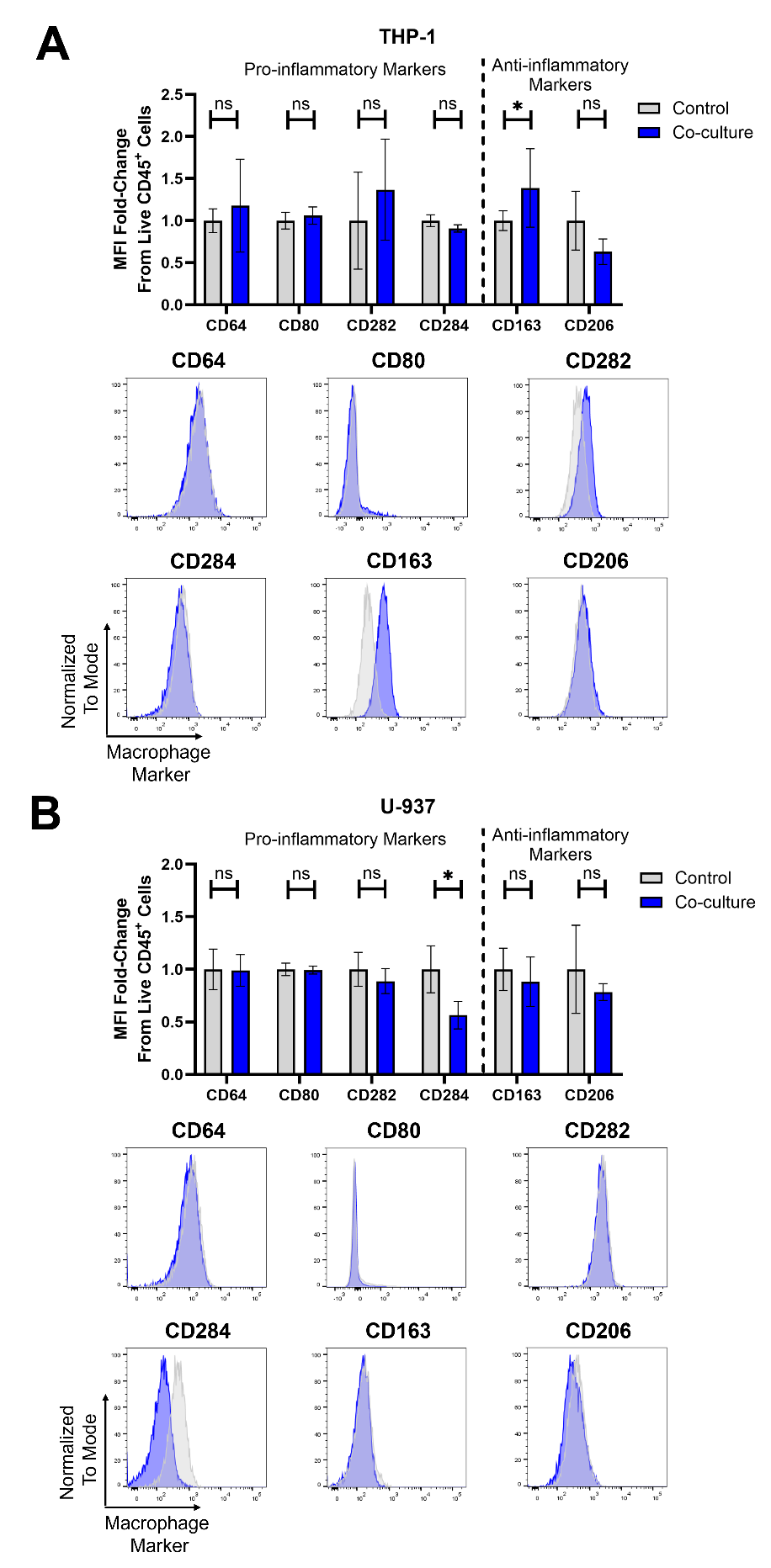


Supplementary Figure 5. Immunophenotyping of THP-1 and U-937 macrophages after co-culture with hDPSCs-1. (A) Fold-change analysis and representative histograms of THP-1 macrophages derived from co-cultures with hDPSCs-1 (Blue) in relation to control THP-1 macrophages (macrophage monocultures - Grey) for the polarization markers CD64 (1.177 ± 0.552 vs 1 ± 0.014, p=0.476); CD80 (1.061 ± 0.103 vs 1 ± 0.098, p=0.501); CD282 (1.369 ± 0.601 vs 1 ± 0.577, p=0.304); CD284 (0.908 ± 0.0439 vs 1 ± 0.069, p=0.136); CD163 (1.389 ± 0.467 vs 1 ± 0.119, p=0.039); and CD206 (0.631 ± 0.151 vs 1 ± 0.348, p=0.202). (B) Fold-change analysis and representative histograms of U-937 macrophages derived from co-cultures with hDPSCs-1 (Blue) in relation to control U-937 macrophages (macrophage monocultures - Grey) for the polarization markers for the polarization markers CD64 (0.989 ± 0.151 vs 1 ± 0.193, p=0.925); CD80 (0.993 ± 0.037 vs 1 ± 0.059, p=0.871); CD282 (0.888 ± 0.119 vs 1 ± 0.160, p=0.237); CD284 (0.563 ± 0.130 vs 1 ± 0.223, p=0.043); CD163 (0.883 ± 0.235 vs 1 ± 0.200, p=0.039); and CD206 (0.784 ± 0.081 vs 1 ± 0.419, p=0.430). This figure represents two independent experiments performed in triplicate. The t-Student test was used to evaluate the immune phenotyping by flow cytometry with 5% of significance level. Legend: ns = non-significant.

|  |  |
| --- | --- |


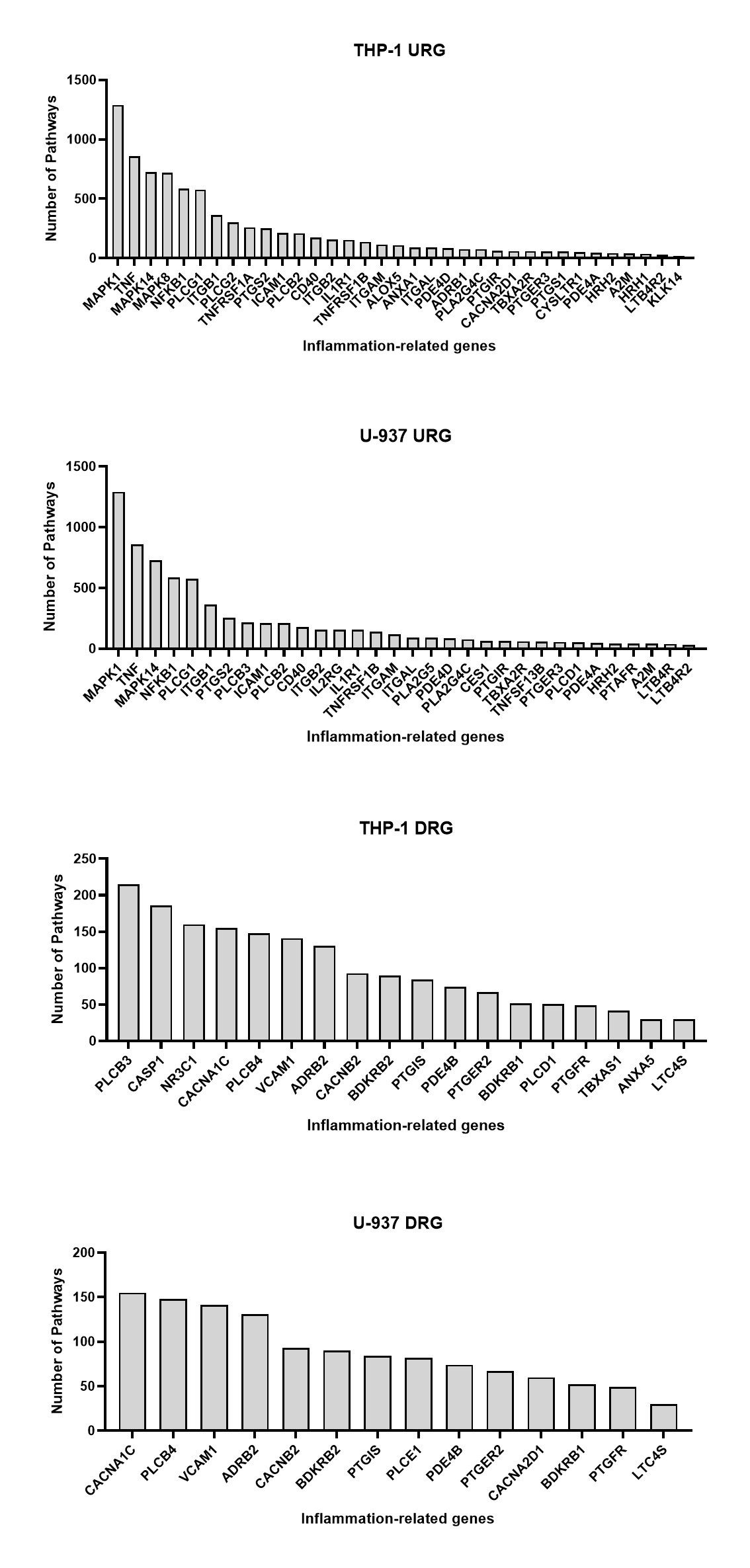


Supplementary Figure 6. Number of Pathways regulated by inflammation-related DEG on hDPSCs after co-culture with THP-1 or U-937 macrophages.

SUPPLEMENTARY TABLES

Supplementary Table 1. qPCR cycling to evaluation of mRNA relative expression.

| PCR cycling step | Temperature | Time | Number of cycles |
| --- | --- | --- | --- |
| Enzimatic Activation | 95ºC | 10 min | 1 |
| Denaturation | 95ºC | 15 seg | 40 |
| Anneling/Extension | 60ºC | 1min |  |

Manufactures proposed Cycling

**Supplementary Table 2.** qPCR Primer sequences

| Gene | Primer Orientation | Primer sequence |
| --- | --- | --- |
| *IL1B* | Forward | 5’-TGGCTTATTACAGTGGCAATGAGGATG-3’ |
|  | Reverse | 5’-TGTAGTGGTGGTCGGAGATTCGTAG-3’ |
| *IL6* | Forward | 5’-CCACTCACCTCTTCAGAACG-3’ |
|  | Reverse | 5’-CATCTTTGGAAGGTTCAGGTTG-3’ |
| *IL10* | Forward | 5’-AAGCCTGACCACGCTTTCTA-3’ |
|  | Reverse | 5’-ATGAAGTGGTTGGGGAATGA-3’ |
| *IL1RN* | Forward | 5’-ATGGAGGGAAGATGTGCCTGTC-3’ |
|  | Reverse | 5’-GTCCTGCTTTCTGTTCTCGCTC-3’ |
| *TNF* | Forward | TGGCCAATGGCGTGGAGCTG |
|  | Reverse | GTAGGAGACGGCGATGCGGC |
| *CXCL10* | Forward | 5’-CCAGAATCGAAGGCCATCAA-3’ |
|  | Reverse | 5’-CATTTCCTTGCTAACTGCTTTCAG-3’ |
| *HPRT1* | Forward | 5’-CCTGGCGTCGTGATTAGTGAT-3’ |
|  | Reverse | 5’-AGACGTTCAGTCCTGTCCATAA-3’ |

**Supplementary Table 3.** Variability measures of multiplex cytokine data

|  |  | **Cytokines (pg/mL); Mean ± SD** | | | | | |
| --- | --- | --- | --- | --- | --- | --- | --- |
| **Cell lineage** | **Group** | **TNF** | **IL-10** | **IL-1b** | **IL-1RA** | **IL-6** | **CXCL10** |
| **THP-1** | Control M0 Macrophages | 15.28 ± 6.018 | 11.62 ± 3.307 | 26.89 ± 9.451 | 21596 ± 11877 | 12.48 ± 21.61 | 43.70 ± 14.16 |
|  | M0 Macrophages + hDPSCs-1 | 12.73 ± 2.259 | 82.85 ± 6.902 | 87.16 ± 4.785 | 22003 ± 7018 | 62226 ± 2533 | 1508 ± 652.3 |
|  | M0 Macrophages + hDPSCs-2 | 4.800 ± 1.509 | 116.4 ± 18.59 | 115.8 ± 10.14 | 21252 ± 6655 | 60529 ± 3516 | 50.69 ± 51.13 |
|  | M0 Macrophages + hDPSCs-3 | 13.84 ± 1.318 | 34.40 ± 3.487 | 74.03 ± 3.012 | 20559 ± 4966 | 53718 ± 3015 | 310.3 ± 72.23 |
| **U-397** | Control M0 Macrophages | 1496 ± 449.6 | 224.4 ± 22.09 | 540.8 ± 93.13 | 20647 ± 941.5 | 118.0 ± 44.83 | 258.1 ± 47.82 |
|  | M0 Macrophages + hDPSCs-1 | 133.3 ± 30.87 | 185.5 ± 32.90 | 699.3 ± 81.05 | 38345 ± 7940 | 63294 ± 4548 | 528.8 ± 108.5 |
|  | M0 Macrophages + hDPSCs-2 | 90.77 ± 8.086 | 151.7 ± 14.73 | 811.7 ± 57.94 | 42488 ± 10985 | 63963 ± 2347 | 151.2 ± 9.022 |
|  | M0 Macrophages + hDPSCs-3 | 269.8 ± 52.83 | 167.6 ± 20.69 | 735.1 ± 103.3 | 42918 ± 17313 | 70139 ± 14254 | 208.2 ± 4.861 |
| **hDPSCs** | hDPSCs-1 | 2.000 ± 0.815 | 0.370 ± 0.246 | 0.0 ± 0.0 | 6.567 ± 11.37 | 2475 ± 220.2 | 11.59 ± 4.486 |
|  | hDPSCs-2 | 0.834 ± 0.313 | 0.413 ± 0.563 | 0.0 ± 0.0 | 35.03 ± 38.93 | 1488 ± 114.1 | 0.227 ± 0.393 |
|  | hDPSCs-3 | 0.867 ± 0.818 | 0.383 ± 0.115 | 0.0 ± 0.0 | 70.47 ± 121.7 | 3325 ± 97.87 | 0 ± 0 |

**Supplementary Table 4.** Variability measures of cytokine qPCR data

|  |  | **Cytokine qPCR Fold-Change; Mean ± SD** | | | | | | | | | | | | |
| --- | --- | --- | --- | --- | --- | --- | --- | --- | --- | --- | --- | --- | --- | --- |
| **Group** | **Sample** | ***TNF*** | **p-value** | ***IL10*** | **p-value** | ***IL1B*** | **p-value** | ***IL1RN*** | **p-value** | ***IL6*** | **p-value** | ***CXCL10*** | **p-value** |  |
| **THP-1** | **THP-1 Macrophages Monoculture** | 1 ± 0.0610 | 0.113 | 1 ± 0.178 | <0.001 | 1 ± 0.021 | <0.001 | 1 ± 0.021 | 0.114 | 0 ± 0 | 0.007 | 1 ± 0.129 | 0.014 |  |
|  | **THP-1 Mactophages co-culture** | 1.10 ± 0.0635 |  | 2.931 ± 0.193 |  | 2.175 ± 0.055 |  | 1.067 ± 0.047 |  | 0.559 ± 0.081 |  | 0.592 ± 0.106 |  |  |
|  | **hDPSCs-1 Monoculture** | 0.0033 ± 0.0015 | 0.135 | 0.046 ± 0.053 | 0.016 | 0.001 ± 0.00 | 0.002 | 0.001 ± 0.00 | 0.006 | 1.001 ± 0.070 | 0.027 | 0.028 ± 0.005 | <0.001 |  |
|  | **hDPSCs-1 from co-culture** | 0.0267 ± 0.0167 |  | 0.459 ± 0.121 |  | 0.067 ± 0.005 |  | 0.019 ± 0.003 |  | 730.9 ± 211.4 |  | 3.637 ± 0.061 |  |  |
| **U-937** | **U-937 Macrophages Monoculture** | 1 ± 0.014 | 0.008 | 1 ± 0.105 | 0.049 | 1 ± 0.102 | 0.026 | 1 ± 0.078 | 0.008 | 0.226 ± 0.080 | 0.004 | 1 ± 0.053 | 0.564 |  |
|  | **U-937 Macrophages co-culture** | 2.648 ± 0.259 |  | 1.226 ± 0.048 |  | 1.352 ± 0.134 |  | 1.609 ± 0.147 |  | 0.611 ± 0.047 |  | 1.083 ± 0.206 |  |  |
|  | **hDPSCs-1 Monoculture** | 0.004 ± 0.001 | 0.104 | 0.001 ± 0.002 | 0.270 | 0.001 ± 0.000 | 0.003 | 0.001 ± 0.000 | 0.059 | 1.001 ± 0.070 | 0.010 | 0.003 ± 0.005 | <0.001 |  |
|  | **hDPSCs-1 from co-culture** | 0.070 ± 0.040 |  | 0.001 ± 0.000 |  | 0.091 ± 0.009 |  | 0.024 ± 0.011 |  | 971.3 ± 170.2 |  | 1.152 ± 0.053 |  |  |
